# Desafíos en la eliminación de las enfermedades tropicales desatendidas en América Latina y el Caribe

**DOI:** 10.7705/biomedica.8149

**Published:** 2025-09-22

**Authors:** Rubén Santiago Nicholls

**Affiliations:** 1 Investigador emérito, Instituto Nacional de Salud, Bogotá, D. C., Colombia rsnichollso@gmail.com Instituto Nacional de Salud Bogotá D. C Colombia rsnichollso@gmail.com

Las enfermedades tropicales desatendidas constituyen un grupo heterogéneo de más de veinte condiciones que afectan principalmente a personas en comunidades empobrecidas y marginadas, predominantemente en regiones tropicales y subtropicales ^1^. Estas enfermedades contribuyen de manera significativa, en su conjunto, a la carga global de morbilidad debido a su carácter crónico y con frecuencia incapacitante, a menudo con secuelas irreversibles, aunque la mortalidad por ellas no suele ser alta. Además, algunas enfermedades tropicales desatendidas se asocian con desfiguración o discapacidades visibles, lo que conduce injustificadamente a que otras personas estigmaticen a quienes las padecen, además de sufrir de discriminación, aislamiento, exclusión social y compromiso de la salud mental.

El término *desatendidas* se acuñó para referirse a un grupo de enfermedades tropicales que reciben poca atención e inversión para el desarrollo de métodos innovadores de diagnóstico, nuevos medicamentos y abordajes terapéuticos, así como para su control, en comparación con otras enfermedades transmisibles como la malaria, el VIH/sida y la tuberculosis ^1^^,^^2^. Esto se debe a que afectan principalmente a grupos pobres y vulnerables de la población que carece de la capacidad de pago de costos elevados de pruebas de diagnóstico, medicamentos y otros insumos en salud. Por esta razón, no resulta atractiva la inversión para el sector empresarial privado, ya que no hay un incentivo económico claro para desarrollar soluciones para estas enfermedades. Estas enfermedades tropicales desatendidas afectan a las poblaciones desatendidas y contribuyen a que se perpetúe en ellas un ciclo de resultados educativos deficientes, oportunidades profesionales limitadas y pobreza ^3^.

Las causas de las enfermedades tropicales desatendidas son diversas ^3^^,^^4^. Un grupo importante de ellas son enfermedades parasitarias crónicas, como la de Chagas, las leishmaniasis y las helmintiasis intestinales, la filariasis linfática, la oncocercosis y la esquistosomiasis. En las Américas, estas tres últimas constituyen un legado histórico de la esclavitud, pues fueron introducidas por el tráfico de esclavos, y encontraron en algunas zonas del Nuevo Mundo las condiciones ecológicas propicias para que se estableciera su transmisión y se mantuviera.

También incluyen infecciones bacterianas como la lepra -una causa importante de morbilidad y discapacidad a nivel mundial- y el pian; enfermedades virales agudas, como el dengue, la fiebre de chikunguña y la rabia humana transmitida por perros; algunas enfermedades micóticas, como el micetoma y la cromoblastomicosis, y el envenenamiento por mordeduras de serpientes y animales ponzoñosos.

Las enfermedades tropicales desatendidas imponen cargas económicas sustanciales a los países en desarrollo, que se estima ascienden a miles de millones de dólares estadounidenses anuales en gastos sanitarios directos, pérdida de productividad y reducción de logros socioeconómicos y educativos. Las repercusiones económicas pueden tener efectos profundos y duraderos en las personas afectadas, sus familias y comunidades, perpetuando un ciclo de pobreza, mientras quienes padecen estas enfermedades luchan por mantener un empleo y acceder a la educación ^1^. A nivel mundial, las enfermedades tropicales desatendidas contribuyen a la pérdida de un estimado de 14,5 millones de años de vida ajustados por discapacidad (AVAD) ^5^.

El avanzar hacia el control y la eliminación de las enfermedades tropicales desatendidas requiere un enfoque integral, que incorpore estrategias como el fortalecimiento de la vigilancia, la administración masiva de medicamentos, el diagnóstico y el manejo clínico adecuado de los casos, de la discapacidad y de las secuelas, el incremento de la cobertura de acceso a agua segura y el saneamiento básico, el manejo integrado de vectores, la concientización, educación, movilización social y fomento de la participación comunitaria, así como de acciones intersectoriales desde el enfoque de *Una salud* ^1^^,^^6^ para el abordaje certero de sus factores ambientales y sociales determinantes.

Los gobiernos, las organizaciones internacionales, las organizaciones no gubernamentales y las comunidades juegan un papel fundamental en los esfuerzos colectivos para controlar o eliminar estas enfermedades, mejorando así la salud, el bienestar y la calidad de vida de las poblaciones expuestas al riesgo de las enfermedades tropicales desatendidas.

Latinoamérica enfrenta desafíos persistentes -como altos niveles de desigualdad de ingresos, disparidades sociales y distribución desigual de recursos- arraigados en factores históricos como las estructuras socioeconómicas, la persistencia de muchas necesidades básicas insatisfechas, y el acceso limitado a vivienda adecuada, agua potable, saneamiento básico, higiene ambiental, educación y atención médica.

Según el informe más reciente del Banco Mundial ^7^, en el 2023, el 25 % de los habitantes de la región vivía con ingresos por debajo de la línea de pobreza de los países de ingresos medios-altos de USD$ 6,85 diarios por persona y el 3,9 % vivía en pobreza extrema, es decir, con un ingreso diario menor de USD$ 2,15 por persona.

En el 2020, alrededor de la cuarta parte de los cerca de 654 millones de habitantes de Latinoamérica y el Caribe, carecían de acceso a servicios seguros de agua potable y dos terceras partes de la población carecían de acceso a instalaciones sanitarias gestionadas de manera segura, siendo las brechas de acceso a estos servicios básicos considerablemente mayores en las zonas rurales ^8^, no solo en términos de cobertura, sino también, de calidad y oportunidad. En el 2019, se estimaba que 15,5 millones de personas todavía se veían obligadas a defecar al aire libre en Latinoamérica y el Caribe ^9^.

En el 2019, el Consejo Directivo de la Organización Panamericana de la Salud (OPS) aprobó, mediante la resolución CD57.R7 ^10^, la "Iniciativa de eliminación de enfermedades transmisibles y condiciones relacionadas en la región de las Américas, 2020-2030, política para aplicar un enfoque integrado y sostenible de las enfermedades transmisibles en la Región de las Américas" ^10^.

Esta iniciativa proporciona un marco común y sostenible con líneas de acción priorizadas para orientar y guiar a los países de la Región en su objetivo de eliminar un grupo prioritario de un poco más de treinta enfermedades transmisibles y otros problemas relacionados ^8^. Su visión es la de un futuro libre de la carga de estas enfermedades en las Américas, a más tardar, para el 2030. Entre las enfermedades incluidas con objetivo de eliminación en esta estrategia, se encuentra un grupo significativo de once enfermedades tropicales desatendidas.

En la iniciativa, se proponen cuatro líneas estratégicas de acción:

1. fortalecer la integración de los sistemas de salud y la prestación de servicios de salud;
2. fortalecer los sistemas estratégicos de información y vigilancia de salud;
3. abordar los factores sociales y ambientales determinantes de la salud, y
4. fortalecer la gobernanza, la rectoría y las finanzas.

Dentro de este contexto, los principales desafíos para controlar y, eventualmente, eliminar las enfermedades tropicales desatendidas, incluyen mejorar la cobertura y el acceso a los servicios de salud preventivos y asistenciales, de buena calidad, la atención primaria en salud y las capacidades de prestación de servicios para poblaciones en condición de vulnerabilidad por sus condiciones socioeconómicas, migrantes, personas desplazadas, pueblos indígenas y minorías étnicas. La constante evaluación del desempeño de los programas y los sistemas de vigilancia para hacer el seguimiento del cumplimiento de los objetivos de eliminación de enfermedades, deben ser reforzados. Es esencial reducir las brechas de coordinación dentro de los sistemas de salud, que involucran a múltiples actores, tanto públicos como privados, al igual que mejorar y fortalecer la comunicación entre el sector salud y otros sectores que abordan los factores socioeconómicos y ambientales determinantes de las enfermedades desatendidas. Finalmente, las comunidades afectadas deben involucrarse activamente en los programas y sus intervenciones en las diferentes etapas -planificación, implementación, seguimiento y evaluación- mediante el fomento efectivo de la movilización social y la participación comunitaria.

Los diecisiete Objetivos de Desarrollo Sostenible (ODS) 2016-2030, aprobados por los estados miembros de la Organización de las Naciones Unidas (ONU), incluyen uno específico sobre salud, el ODS 3: "Garantizar una vida sana y promover el bienestar para todos en todas las edades" ^11^, que a su vez incluye la meta 3.3, "Para 2030, poner fin a las epidemias del SIDA, la tuberculosis, la malaria y las enfermedades tropicales desatendidas y combatir la hepatitis, las enfermedades transmitidas por el agua y otras enfermedades transmisibles".

Solo la acción coordinada de los actores relevantes del sector salud con otros múltiples sectores para reducir la pobreza hasta eliminarla y mejorar las condiciones de vida de las poblaciones expuestas al riesgo de enfermedades tropicales desatendidas, permitirá la disminución significativa de las inequidades sociales y de la desatención histórica de los grupos poblacionales vulnerables y, con ella, la reducción sostenida del impacto de las enfermedades tropicales desatendidas en la salud y las condiciones de vida, y su eventual eliminación.
